# Comparison of bronchial responsiveness to ultrasonically nebulized distilled water (UNDW), methacholine, and ultrasonically nebulized distilled cold water (UDCW) in patients with sulfur mustard gas-induced asthma

**DOI:** 10.1080/15563650701382730

**Published:** 2007-06-20

**Authors:** Ali Emad, Yasaman Emad

**Affiliations:** 1 Associate Professor of Medicine, Department of Internal Medicine, Section of Pulmonary Diseases, Shiraz University of Medical Sciences, Shiraz, Iran; 2 Shiraz University, Shiraz, Iran

**Keywords:** Musta Mustard, Asthma, Metacholine, Provocation

## Abstract

**Objective:**

To evaluate bronchial challenges using three different stimuli as screening tools for bronchial hyper-responsiveness in sulfur mustard gas-induced asthma.

**Design:**

Randomized, cross-over clinical study.

**Setting:**

University hospital.

**Patients:**

Eighteen veterans with mustard gas-induced asthma and 18 normal veterans as the control group.

**Intervention:**

Pulmonary function tests and inhalation challenges with ultrasonically nebulized distilled water (UNDW), methacholine, and ultrasonically nebulized cold water (UNDCW) were performed on all patients and subjects.

**Results:**

Six mustard gas-induced asthmatic veterans did not respond to a 20% in FEV_1_ after distilled water (13.3%), and two of them (11.11%) did not respond with distilled cold water; all responded with methacholine. Only one healthy subject developed a PC20 FEV_1_ after methacholine but did not with both distilled water and distilled cold water challenges. The asthmatic patients were sensitive to distilled water with a median PD20 of 7.24 ± 3.83 ml (range 2.54 ml to 15.83 ml), and sensitive to cold water with a median PD20 of 6.42 ± 6.24 ml (range 1.92 ml to 25.15 ml). The median PC20 methacholine was 1.90 ± 1.88 mg/ml (range 0.14 mg/ml to 6.20 mg/ml). In patients with a positive response to the distilled water challenge test, no significant correlation was found between PC20 of methacholine and PD20 of distilled water (Rho = −0.34, p = 0.25), whereas in patients whose responses to distilled cold water (DCW) were positive, PD20 of distilled cold water (DCW) correlated well with PC20 of methacholine (Rho = −0.69, p = 0.006).

**Conclusion:**

Overall, the methacholine challenge test is the best method to distinguish these asthmatic patients from normal subjects in this study. When compared to the methacholine challenge, although the airway response to ultrasonically nebulized distilled cold water test was somewhat less sensitive, it may be used as a simple, fast, inexpensive, and relatively reliable method to predict the absence of asthma in sulfur mustard gas-induced asthma.

## Introduction

Sulfur mustard gas is used as a vesicant chemical warfare agent (1). This chemical gas is an alkylating agent that is acutely toxic to the skin, eyes, and respiratory system (2–4). Upper and lower respiratory tracts may be acutely damaged after its inhalation (3,5,6). The diversity of the effects of sulfur mustard gas inhalation upon the respiratory system has been investigated following a single and heavy exposure in Iranian veterans (2). Bronchial hypersensitivity and asthma may occur as an important chronic sequella of the manifestation of sulfur mustard gas inhalation (2,7).

On the other hand, bronchial hyper-responsiveness is a characteristic of patients with bronchial asthma (19). Methacholine challenge testing is a well-established means of evaluating the degree of airway responsiveness (14,15). Nonpharmacological challenge tests involve provocation with cold air, exercise, and inhalation of isotonic and nonisotonic aerosols (24,26,29). In general, these methods provide a high specificity but somewhat less sensitivity. Since there are no reports on the behavior response of airway hyper-reactivity to a variety of stimuli in asthmatic veterans who have been exposed to an acute and heavy exposure of sulfur mustard gas, this study was carried out to define its pattern. The purpose of the current investigation was to investigate and compare the responses to inhaled methacholine, ultrasonically nebulized distilled water, and ultrasonically nebulized distilled cold water in mustard gas-induced asthma.

## Methods

### Patients with mustard gas-induced asthma

The study population consisted of two groups of non-smoking veterans. The group of asthmatic subjects studied whose exposure to mustard gas has been previously confirmed by studies on their urine and vesicular fluid consisted of 18 non-smoking veterans. They were aged 30–41 years (38.50 ± 3.58). All patients had an absence of preceding respiratory symptoms and pre-exposure asthma. None of the patients had a family history of atopy or asthma. Patients with proven cardiovascular diseases and those with exposure to other environmental or pharmacological agents known to cause extrinsic allergic alveolitis, and cases with evidence of recent infection or exacerbation of their diseases were excluded. Overall, asthma was diagnosed in 21 cases as a late sequela of pulmonary effects of sulfur mustard gas exposure. Three patients were excluded according to our exclusion criteria. The initial pulmonary, skin, and eye symptoms in these victims in 1986 following sulfur mustard gas exposure are seen in Table 1. Baseline characteristics of the asthmatic subjects are presented in Table 2. The diagnosis of asthma was made when they met at least two of the following criteria: 1) diurnal variability in peak expiratory flow (PEF) rate >20% (8,9); 2) the reversibility of FEV_1_ as described by Ries et al. (10); and 3) typical history of attacks of dyspnea, wheezing, or both, nocturnal cough either spontaneously or triggered by irritants, respiratory infections, or exercise.

**Table 1:** Initial pulmonary, eye, and skin symptoms in patients with sulfur mustard gas-induced asthma in 1986

		Number of patients
Pulmonary	Cough	18
	Dyspnea at rest	18
	Chest tightness	16
	Wheeze	0
	Pulmonary infiltration	0
Skin	Blister and bulla	16
	Erythema	16
Eye	Photophobia	15
	Lacrimation	15
	Lid edema	15
	Conjunctivitis	15

**Table 2:** Results of baseline FEV_1_ measurements, PC_20_ (mg/ml), PD_20_ of distilled water (ml), and PD_20_ of distilled cold water (ml) in patients with mustard gas-induced asthma

		Methacholine		UNDW*		UNDCW*	
				
	Age	Baseline FEV_1_, %	PC_20_ (mg/ml)	Baseline FEV_1_, %	PD_20_ (ml)	Baseline FEV_1_, %	PD_20_ (ml)
1	38.	76.4	.14	81.4	2.54	88.1	1.92
2	43.	92.9	3.5	101.9	NR**	90.5	6.58
3	38.	91.5	.49	81.2	10.050	85.3	2.85
4	37.	90.3	2.6	88.1	NR**	78.3	4.19
5	36.	97.1	1.2	88.1	5.77	106.1	3.16
6	44.	94.3	.87	90.3	NR**	94.8	NR**
7	43.	88.5	.40	93.2	9.260	86.7	3.75
8	36.	89.5	5.8	99.7	NR**	85.9	25.15
9	32.	84.2	.83	95.8	6.73	98.6	5.63
10	35.	84.2	.21	87.4	2.71	89.9	4.20
11	33.	82.7	1.9	89.3	4.79	79.5	3.56
12	36.	98.7	.47	90.8	NR**	95.5	NR**
13	44.	93.5	6.2	97.9	15.83	85.1	14.33
14	40.	85.2	.18	96.5	8.15	82.3	2.47
15	41.	76.8	1.6	80.3	NR**	83.9	14.20
16	39.	81.3	2.4	75.4	11.01	89.1	3.91
17	38.	83.5	1.3	80.2	5.67	98.4	2.38
18	40.	86.8	4.12	102.3	4.44	80.2	4.59
Mean ± SD	38.50 ± 3.58	87.63 ± 6.41	1.90 ± 1.88	89.98 ± 8.06	7.24 ± 3.83***	88.78 ± 7.47	6.42 ± 6.24****

* UNDW=nebulized ultrasonic distilled water, UNCDW=nebulized ultrasonic distilled cold water.

** NR=negative response.

*** Mean±SD in those with positive response to DW.

**** Mean±SD in those with positive response to DCW.

All patients in this study used β_2_-agonists inhaled as needed. Seven patients used inhalers beclomethasone diproprionate on a regular basis, and five cases required daily inhaled bronchodilator. None required systemic corticosteriods to control their asthma. None were allowed to receive inhaled β_2_-agonist, inhaled ipratropium bromide for 8 h, theophylline and inhaled beclomethasone for four days prior to the study.

### Normal controls (control group)

Eighteen sex- and age-matched healthy subjects with a mean age of 36.16 ± 5.46 years old, with no history of upper or lower respiratory tract symptoms served as a control group. These healthy male veterans had been in combat zones but not exposed to chemical agents. They had normal pulmonary function parameters. Cases with a family history of asthma or other allergic respiratory disorders were excluded. There was no history of respiratory tract infection during the four weeks prior to the study. Overall, 22 non-sulfur mustard gas-exposed veterans (as the control group) were recruited during a one month period and four of them were excluded according to our exclusion criteria.

### Study design

At the time of the study, all veterans had been free of symptoms of any respiratory illness for four weeks. The FEV_1_ was more than 70% of predicted normal at entry of the study.

The study protocol consisted of inhalation challenges with ultrasonically nebulized distilled water, methacholine, and ultrasonically nebulized cold water. The challenges were performed at the same time of day for each subject, within 10 days and at least two days apart. The persons administrating the intervention were blinded to the group assignment. The study was a randomized, cross-over study. Normal subjects and patients were randomly assigned to start with either ultrasonically nebulized distilled water, methacholine, or ultrasonically nebulized distilled cold water.

All cases were initially examined at time of entry to our pulmonary lab. They rested for 30 minutes before testing. All cases signed an informed written consent and had a complete history and physical examination.

### Measurement of pulmonary function

Pulmonary function tests were performed at each visit. These tests were measured through spirometric assessment according to the standards advocated by the American Thoracic Society (11). An experienced physician conducted all spirometric measurements for all subjects using FUDAC 50 (Fukunda Sangyo Co., LTD, Japan). Each patient was well trained to give his best effort. Results were expressed as percentage-predicted based on accepted reference standards (12,13). The highest values were chosen and reported.

### Challenge with ultrasonically nebulized distilled water (UNDW)

Aerosols of room-temperature distilled water were generated by an ultrasonic nebulized (Heyer Orion 1, BAD EMS). Its mean output was 2.40 ml/min. Oral inhalation was ensured by using nose clips. Following baseline spirometric measurements, the subjects were instructed to inhale the mist from a facemask at tidal breathing (15 to 20 times/min). Inhalation of the aerosol was initiated with 1.3 ml. The patients inhaled increasing volumes of UNDW until FEV_1_ was reduced by 20% from baseline value, or the water dose exceeded 34.8 ml (14,15). The cumulated dose of delivered nebulized water producing a 20% fall in FEV_1_ (PD_20_ UNDW) was calculated by linear interpolation on the dose-response curve.

### Challenge with methacholine

The aerosols of methacholine was generated from a DeVil-biss no 45 nebulized operated by compressed air at 50 ib/in^2^ and a flow rate of 5 l/min to give an output of 0.156 ml/min. They were delivered through the mouth while the subjects were wearing a nose clip by five slow vital capacity maneuvers, each separated by a five second breath hold. At first, the subjects were asked to inhale from a phosphate buffered saline. Then, the aerosols of methacholine solutions were nebulized at five minute intervals by a two-fold-increasing concentration of methacholine (0.03 − 25 mg/ml). Spirometric values were determined before and 0.5 and 1.5 minutes after each dose. The challenge was stopped after reaching the concentration of methacholine that provoked a 20% reduction in forced expiratory volume in one second (FEV_1_) from pre-challenge baseline (PC_20_ M). The provocative concentrations of methacholine required to produce a 20% fall in FEV_1_ from the post-saline FEV_1_ (PC20) was calculated by interpolation.

### Challenge with ultrasonically nebulized distilled cold water (UNDCW)

The method of this challenge test was similar to provocation with ultrasonically nebulized distilled water, as previously described. In this procedure, the water canister was filled with cold water (4°C). A constant water temperature was maintained by adding ice cubes to the canister after each challenge test. The patients inhaled increasing volumes of UNCDW until FEV_1_ was reduced by 20% from baseline value or the water dose exceeded 34.8 ml. The provocative cumulated dose of delivered cold nebulized water producing a 20% fall in FEV_1_ (PD_20_ UNCDW) was calculated by linear interpolation on the dose-response curve.

## Statistics

All results are mean ± SD unless otherwise indicated. Comparisons between the study groups were performed using the Mann–Whitney U test. Correlations between different parameters were determined by Spearman's rank correlation coefficient. Logarithmic transformations were applied to all values for PD_20_ UNDW, PC_20_ M, and PD_20_ UNDCW before comparison. p values of less than 0.05 were regarded as significant.

## Results

The initial pulmonary, skin, and eye symptoms in these victims in 1986 following sulfur mustard gas exposure are seen in Table 1. All mustard-gas induced asthmatic patients in this study who survived a short-term and massive sulfur mustard gas exposure recovered within a few weeks in 1986. However, they developed episodic wheeze, breathlessness, chest tightness, or cough and variable airflow obstructive pattern in pulmonary function tests spontaneously or from many and varied environmental stimuli following the initial gas exposure. All had a diurnal variability in PFT rate. A reversibility of FEV_1_ was also noted in all subjects of the group of asthma.

There was no significant difference between the mean age of the control subjects (36.16 ± 5.46) with the asthmatic group (38.50 ± 3.58) (p = 0.18). The results of the challenge tests for the asthmatic veterans are summarized in Table 2. Baseline FEV_1_ measurements (as percent predicted) were not significantly different between the normal subjects and asthmatics at the beginning of all challenge tests. No significant difference between baseline FEV_1_ (as percent predicted) on the three challenge days was found (p = 0.12, 0.2, 0.08, respectively).

Six mustard gas-induced asthmatic veterans did not sustain a 20% in FEV_1_ after ultrasonically nebulized distilled water (33.3%), and two of them (11.11%) did not with ultrasonically nebulized distilled cold water; all did with methacholine. In other words, all mustard gas-induced asthmatics were sensitive to the methacholine challenge test. Sixteen patients were sensitive to the UNDCW challenge test, but only 12 patients responded to ultrasonically nebulized distilled water (UNDW).

Only one healthy subject developed a PC20 FEV_1_ after methacholine (PC20 methacholine = 18.25 mg/ml). The PC20 methacholine in the rest of healthy cases was >25 mg/ml. No control group responded to both ultrasonically distilled water and ultrasonically distilled cold water challenges.

The asthmatic patients were sensitive to distilled water with a median PD20 of 7.24 ± 3.83 ml (range 2.54 ml to 15.83 ml) and sensitive to cold water with a median PD20 of 6.42 ± 6.24 ml (range 1.92 ml to 25.15 ml). The median PC20 methacholine was 1.90 ± 1.88 mg/ml (range 0.14 mg/ml to 6.20 mg/ml). No correlation between PC20 of methacholine and baseline FEV_1_ was seen (Rho = −0.17, p = 047).

**Fig. 1:**
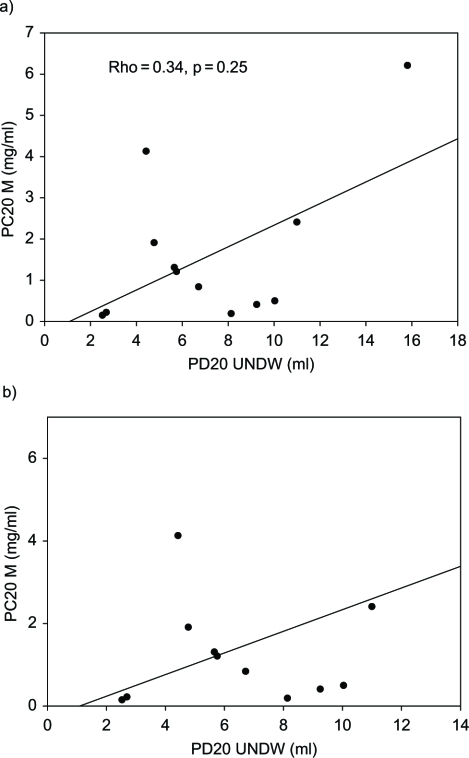
A) Comparison of responses to provocative concentration of methacholine causing a 20% fall in FEV_1_ (PC20 M) and provocative dose of distilled water causing a 20% fall in FEV_1_ (PD20 UNDW) values in patients with a positive response to ultrasonically nebulized distilled water (UNDW). B) Comparison of PC20 M and PD20 UNDW values in patients with PC20 ≤ 6 mg/ml and PD20 UNDW ≤ 12 ml.

**Fig. 2:**
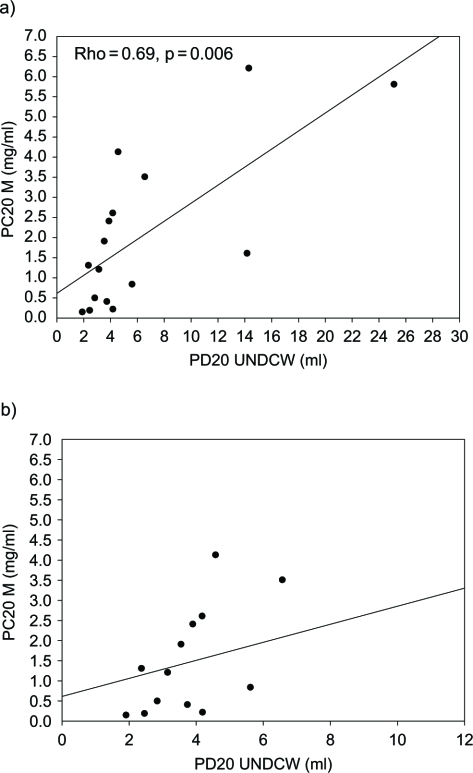
A) Comparison of responses to provocative concentration of methacholine causing a 20% fall in FEV_1_ (PC20 M), and provocative dose of distilled cold water causing a 20% fall in FEV_1_ (PD20 UNDCW) values in patients with a positive response to ultrasonically nebulized distilled cold water (UNDCW). B) Comparison of PC20 M and PD20 UNDCW values in patients with PC20 ≤ 8 mg/ml and PD20 UNDCW ≤ 12 ml.

In patients with a positive response to the distilled water challenge test, no significant correlation was found between PC20 of methacholine and PD20 of distilled water (Rho = −0.34, p = 0.25) (Fig. 1).I In patients whose responses to distilled cold water (UNDCW) were positive, PD20 of distilled cold water (UNDCW) correlated well with PC20 of methacholine (Rho = −0.69, p = 0.006) (Fig. 2).

## Discussion

Sulfur mustard gas may shed the columnar cells of the epithelial lining of the upper respiratory tract in an acute heavy exposure. This event may be accompanied by peribronchial edema, hyperemia of the blood vessels, cellular infiltrations in the submucosa, and serious vacuolization and disorganization of cytoplasma and nuclear structures (16,17). Bronchial hypersensitivity and asthma may be due to the direct consequence of these cytotoxic and inflammatory effects of this toxic gas (18).

Airway hyper-responsiveness is a characteristic and key feature of asthma (19). Clinically, and for research purposes, responsiveness to an administered drug or compound remains the most useful physiological test in the assessment of asthma (20). In this study, we have evaluated bronchial challenge using three different stimuli as screening tools for bronchial hyper-responsiveness in sulfur mustard gas-induced asthma for the first time. The obtained data were compared.

The applied tests in this study (methacholine and UNDCW) were shown to have accepted sensitivity screening tests in the selected mustard-gas induced asthma. It is obvious that these tests (alone) will discriminate between the other consequences of sulfur mustard gas exposition such as chronic bronchitis, fibrosis, and bronchiectasis. Additional interventions such as BAL, computed tomographic (CT) scan of the chest, pulmonary function test with restrictive ventilatory defect, diffusing capacity of carbon monoxide (Dlco), and histological findings will be helpful for the diagnosis of other consequences of sulfur mustard gas exposition, like pulmonary fibrosis and bronchiectasis.

In our study, all mustard gas-induced asthmatic patients were sensitive to methacholine (100%). This study has shown that bronchial hyper-responsiveness to methacholine has no correlation with baseline % FEV_1_ values in patients with sulfur mustard-gas asthma. Overall, the methacholine challenge test was the best method to distinguish these asthmatic patients from the normal subjects in this study. This suggestion is not different from the other non-mustard gas-induced asthmatic cases with other studies (21,22).

Only 12 subjects (66.7%) from all mustard gas-induced asthmatics were sensitive to ultrasonically nebulized distilled water (DW). Therefore, the sensitivity of the airway response to UNDW challenge (66.7%) is relatively low as compared to that of methacholine (100%). No significant correlations were found between PC20 of methacholine and PD20 of distilled water (Rho = −0.34, p = 0.25). This finding may indicate that UNDW may act via a different pathway or mechanism to cause bronchoconstriction in our patients as compared to methacholine challenge. These findings are similar to other previous reports about non-mustard gas-induced asthma (21).

Interestingly, 16 of 18 cases of mustard gas-induced asthmatics were sensitive to ultrasonically nebulized distilled cold water (UNDCW). There was also a significant correlation between PC20 of methacholine and PD20 of distilled cold water (Rho = −0.56, p = 0.02). It is evident that sensitivity for UNDCW (88.9%) was less than that for methacholine (100%) in revealing airway hypersensitivity in sulfur mustard gas-induced asthma. The data showed that the positive and negative predictive values of UNDCW for diagnosis or exclusion of mustard gas-induced asthma were 100% in this study. This challenge test was well tolerated by all patients, whereas three patients had mild side effects with methacho-line (increase water secretion, asthmatic attack).

This study verifies that ultrasonically nebulized distilled cold water (UNDCW) challenge is a potent stimulus to cause airway narrowing (23–26). Other studies believe that cold and dry air may cause airway narrowing through the release of leukotrienes (27). Airway smooth muscle can be constricted directly by agonists, such as methacholine or histamine, which activate receptors on the smooth-muscle cells, or by indirect mechanisms, such as cold air or exercise, which, at least in part, induce the release of bronchoactive mediators from mast cells (28,29).

In conclusion, as compared to methacholine challenge, although the airway response to the UNDCW test was somewhat less sensitive, our results demonstrate that it seems that UNDCW may be used as a simple, fast, inexpensive (the cost of the use of the methacholine test is about $100, whereas the UNDCW test is about $30 in Iran), and relatively reliable method to predict the absence of asthma in sulfur mustard gas-induced asthma.
